# Close of Lecture Term, in the Medical Department of the University of Buffalo

**Published:** 1854-03

**Authors:** 


*Close of Lecture Term in the Medical Department of the University of Buffalo.*—Just as we write, most of the various medical colleges in our country are closing the labors of the season. To-day (22d Feb.) many hundreds of students have received their diplomas, and been sent out into the world of sickness, to fulfill their mission of healing.

The three years of student life, with their immunities from responsibility and care, their pleasant social intercourse, and their buoyant hopes, are here brought up against the dead-wall of a diploma. The transition state is over: the facts gathered, the theories received, are to come to the test of action. Few of those who have this day become M. D., can look forward with any kind of certainty to the path before them. Over it hangs a vail of mist, which conceals all distant objects, and only lifts as they advance, to show them the next step in their onward progress.

Most graduating students look sad upon commencement day. There is a feeling of doubt and unrest which troubles their hearts, and casts a shade over the hour to which they had looked forward with joyful anticipation. They have a problem to be solved—the great problem of every man's life—the question of their own success and usefulness. In the hurry and excitement of the lecture course it has been forgotten; but now it comes to them as a new thing, a sort of puzzle, a quiz for which they are not prepared, and which no grinding can enable them to meet.

Patience and endeavor alone can solve it. Work and study, life's hard realities, the emergencies of practice, the struggles of competition, must be the touchstones of character. H.

